# Oligomers of the lipodystrophy protein seipin may co-ordinate GPAT3 and AGPAT2 enzymes to facilitate adipocyte differentiation

**DOI:** 10.1038/s41598-020-59982-5

**Published:** 2020-02-24

**Authors:** M. F. Michelle Sim, Elisa Persiani, Md. Mesbah Uddin Talukder, George D. Mcilroy, Ahlima Roumane, J. Michael Edwardson, Justin J. Rochford

**Affiliations:** 1 University of Cambridge Metabolic Research Laboratories, Institute of Metabolic Science, Addenbrooke’s Hospital, Cambridge, CB2 0QQ UK; 2 Rowett Institute and the Aberdeen Cardiovascular and Diabetes Centre, University of Aberdeen, Foresterhill, Aberdeen AB25 2ZD UK; 3 Department of Pharmacology, University of Cambridge, Cambridge, CB2 1PD UK

**Keywords:** Mechanisms of disease, Type 2 diabetes

## Abstract

Seipin deficiency causes severe congenital generalized lipodystrophy (CGL) and metabolic disease. However, how seipin regulates adipocyte development and function remains incompletely understood. We previously showed that seipin acts as a scaffold protein for AGPAT2, whose disruption also causes CGL. More recently, seipin has been reported to promote adipogenesis by directly inhibiting GPAT3, leading to the suggestion that GPAT inhibitors could offer novel treatments for CGL. Here we investigated the interactions between seipin, GPAT3 and AGPAT2. We reveal that seipin and GPAT3 associate via direct interaction and that seipin can simultaneously bind GPAT3 and AGPAT2. Inhibiting the expression of seipin, AGPAT2 or GPAT3 led to impaired induction of early markers of adipocyte differentiation in cultured cells. However, consistent with normal adipose mass in GPAT3-null mice, GPAT3 inhibition did not prevent the formation of mature adipocytes. Nonetheless, loss of GPAT3 in seipin-deficient preadipocytes exacerbated the failure of adipogenesis in these cells. Thus, our data indicate that GPAT3 plays a modest positive role in adipogenesis and argue against the potential of GPAT inhibitors to rescue white adipose tissue mass in CGL2. Overall, our study reveals novel mechanistic insights regarding the molecular pathogenesis of severe lipodystrophy caused by mutations in either seipin or AGPAT2.

## Introduction

Individuals with loss-of-function mutations in the *BSCL2* gene, encoding the protein seipin, suffer from congenital generalised lipodystrophy type 2 (CGL2) and severe metabolic disease^1,2^. This phenotype appears to result at least in part from a reduced capacity to develop adipocytes from precursor cells as well as a failure to maintain functional mature adipocytes^3–10^. Seipin is highly conserved evolutionarily; it is principally localised to the endoplasmic reticulum (ER) membrane and forms highly organised homo-oligomers^11–15^. This has led to investigation of seipin as a potential scaffold protein and the identification of several seipin-binding proteins involved in lipid droplet biology and adipocyte development and function^16^. We previously showed that seipin can simultaneously bind to both AGPAT2 and lipin 1, key pro-adipogenic enzymes that act sequentially in the same lipid biosynthetic pathway^17,18^. A more recent study reported that seipin can also bind to GPAT enzymes, which catalyse the step immediately preceding that catalysed by AGPAT2^19^. The authors demonstrated that seipin can act as an inhibitor of the glycerol-3-phosphate acyltransferase activity of GPAT3 and proposed that the loss of this inhibition leads to impaired adipocyte differentiation when seipin is absent^19^. Moreover, they reported that pharmacological inhibition of GPAT3 could partly rescue adipogenesis in seipin-deficient preadipocytes and so might offer a potential therapy for the treatment of patients with CGL2^19^. However, these data partly contradict a previous study showing that GPAT3 positively regulates adipogenesis in cultured preadipocytes^20^, leading us to examine this issue further. In this study we confirm the observation that seipin binds GPAT3 and use atomic force microscopy (AFM) to reveal that this involves direct contact between the two proteins. Moreover, we find that seipin can increase the interaction between GPAT3 and AGPAT2, suggesting that seipin oligomers can scaffold these two proteins in a single complex. However, we did not find that inhibition of GPAT3 could rescue adipogenesis in cells lacking seipin, nor that increased GPAT3 expression impaired adipocyte development. Our results, therefore, question the notion that inhibition of GPAT3 could offer a potential therapeutic option for the treatment of CGL2.

## Results

### Seipin can associate with GPAT3 in developing adipocytes

**Figure 1:**
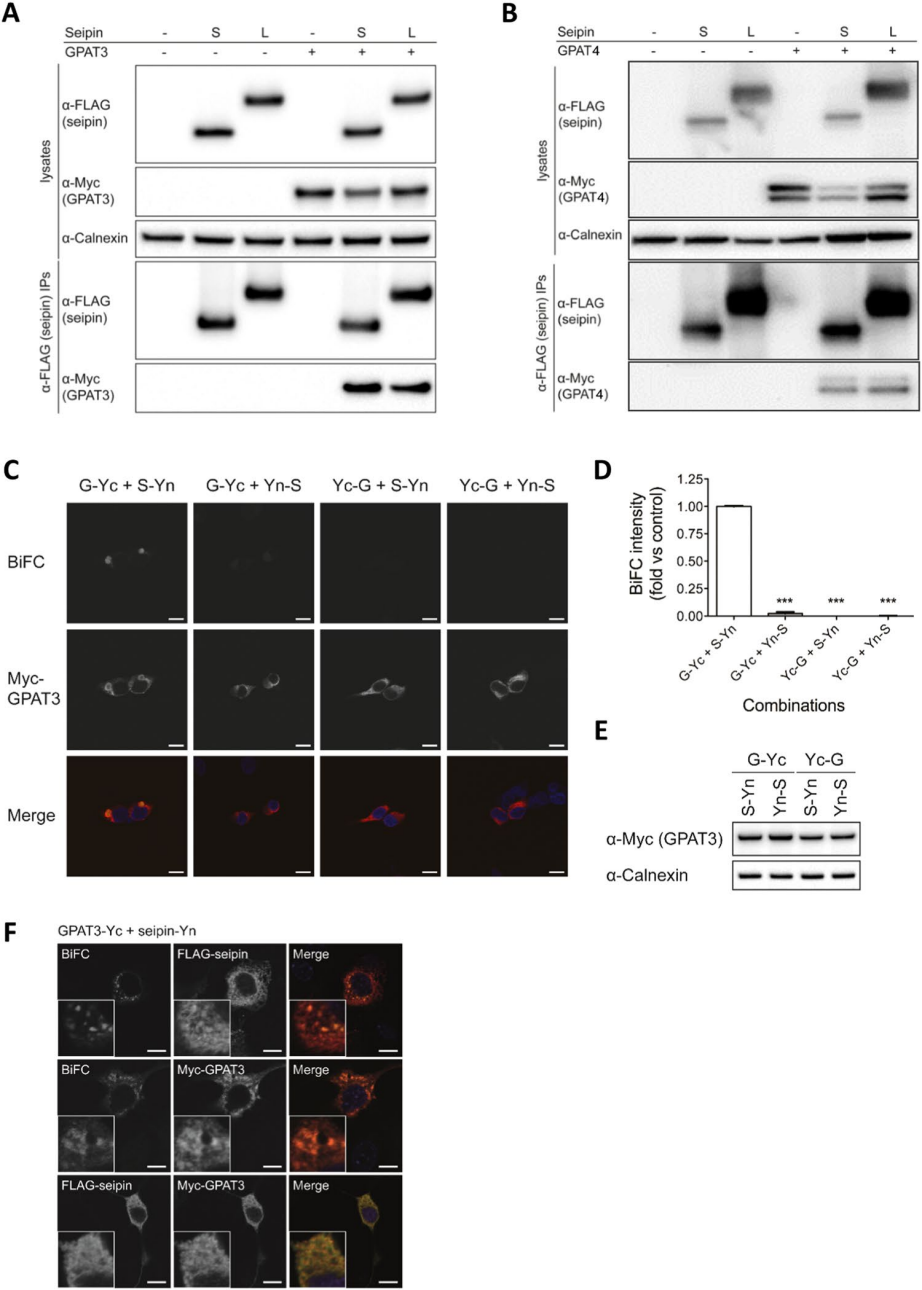
Seipin can associate with GPAT3 and GPAT4. HEK293 cells were transfected with GPAT3-Myc (**A**) or GPAT4-Myc (**B**) in the absence or presence of either the short (S) or long (L) forms of FLAG-tagged seipin. Lysates and anti-FLAG immunoprecipitates were analysed by SDS-PAGE and immunoblotting with antibodies to FLAG or Myc to detect tagged proteins or calnexin as a loading control. (**C**) HEK293 cells were transfected with constructs in which the N-terminal fragment of YFP was fused to the C or N terminus of seipin (S-Yn or Yn-S, respectively), and constructs in which the C-terminal fragment of YFP was fused to either the C or N terminus of GPAT3-Myc (G-Yc and Yc-G, respectively). A temperature shift was used to induce the formation of reconstituted YFP then cells were fixed and stained for GPAT3-Myc (red) and DAPI to label nuclei. The presence of a YFP signal (yellow) indicates an interaction between GPAT3 and seipin. Scale bars, 10 μm. (**D**) Quantified BiFC signal intensity. Errors are SEM (n = 3). ^***^Indicates a difference in YFP fluorescence compared with G-Yc/S-Yn (p < 0.001). (**E**) Anti-Myc immunoblot showing GPAT3-Yc and Yc-GPAT3 expression and calnexin as a loading control. (**F**) 3T3-L1 preadipocytes were co-transfected with FLAG-seipin-Yn and Myc-GPAT3-Yc immediately after the induction of differentiation. Three days later cells were fixed and anti-FLAG or anti-Myc antibodies were used to detect FLAG-seipin-Yn or Myc-GPAT3-Yc, respectively. Identically transfected cells were co-immunostained for FLAG-seipin-Yn and Myc-GPAT3-Yc (lower panels). However these latter cells were not temperature shifted so as to prevent the formation of YFP which would otherwise confound the use of Alexa Fluor488 labelled secondary antibodies in order to detect the FLAG epitope. Scale bars, 10 μm.

To confirm the reported interaction of seipin with GPAT enzymes and examine whether either the short or long form of seipin preferentially binds these enzymes, we co-expressed FLAG-tagged human seipin with either Myc-tagged human GPAT3 (Fig. 1A) or Myc-tagged human GPAT4 (Fig. 1B). GPAT3 or GPAT4 could clearly be detected in anti-FLAG immunoprecipitates using either the long or short forms of seipin, demonstrating that both GPAT enzymes can interact with either isoform of seipin. Previous studies have indicated that GPAT3 may constitute the more significant microsomal GPAT activity in adipocytes and have a greater role than GPAT4 in the differentiation of 3T3-L1 adipocytes^20,21^. Meanwhile, GPAT4 may play important roles in regulating lipid metabolism and insulin sensitivity in the liver^22^. In similar experiments using C3H10T1/2 cells undergoing adipogenesis, we specifically inhibited the expression of either GPAT3 (Fig. S1A) or GPAT4 (Fig. S1B) using siRNA. GPAT3 knockdown was more effective than GPAT4 knockdown at inhibiting the expression of key markers of adipogenesis, including PPARγ and C/EBPα (Fig. S1C,D). In light of these data and previously published work, we focussed on the interaction of seipin with GPAT3 as this is likely to be most relevant to the regulation of early adipogenesis.

As we had previously reported the interaction of seipin with AGPAT2^18^, we also compared the binding of seipin to either GPAT3 or AGPAT2. We typically observed slightly higher expression of AGPAT2 *versus* GPAT3 in these experiments making accurate, direct comparison difficult. However, overall we observed that the capacity of seipin to interact with either protein appeared similar (Fig. S1E)

To further validate the interaction between GPAT3 and seipin we turned to bimolecular fluorescence complementation (BiFC) analysis. The C-terminal portion of YFP (Yc) was fused to either the N or C terminus of GPAT3 to generate Yc-GPAT3 or GPAT3-Yc, respectively. Similarly, the N-terminal portion of YFP (Yn) was fused to the N or C terminus of seipin to generate Yn-seipin and seipin-Yn, respectively. Co-expression of GPAT3-Yc and seipin-Yn led to YFP fluorescence within cells, indicating an association between GPAT3 and seipin, (Fig. 1C,D). In contrast we detected no YFP fluorescence when cells were transfected with other combinations of seipin and GPAT3 fusion proteins, despite similar GPAT3 expression (Fig. 1C–E). The specific requirement for a particular orientation of half-YFP proteins implies a specific interaction between seipin and GPAT3 rather than any non-specific aggregation of these proteins. These data confirm the previous report that seipin and GPAT3 can interact^19^ using a different technique to analyse this interaction in intact cells.

We next examined the interaction between seipin and GPAT3 in developing adipocytes by co-transfecting 3T3-L1 preadipocytes with seipin-Yn and GPAT3-Yc before inducing the cells to differentiate. Reconstituted YFP fluorescence could be clearly observed in these cells at day 3 of differentiation. The BiFC signal co-localized substantially with either GPAT3 or seipin expressed in the cells (Fig. 1F). We also transfected cells in an identical manner but omitted the temperature shift which is required to generate YFP fluorescence in BiFC assays. This revealed significant co-localization of the seipin-Yn and GPAT3-Yc proteins (Fig. 1F, lower panels). We observed no fluorescent BiFC signal when seipin-Yn was co-expressed with Yc-GPAT3 in differentiating 3T3-L1 adipocytes, again indicating that the association between seipin and GPAT3 in developing adipocytes occurs with a defined orientation (Fig. S1F,G).

### Seipin binds GPAT3 via its evolutionarily conserved core domains whilst pathogenic mutations in seipin can affect this interaction

**Figure 2:**
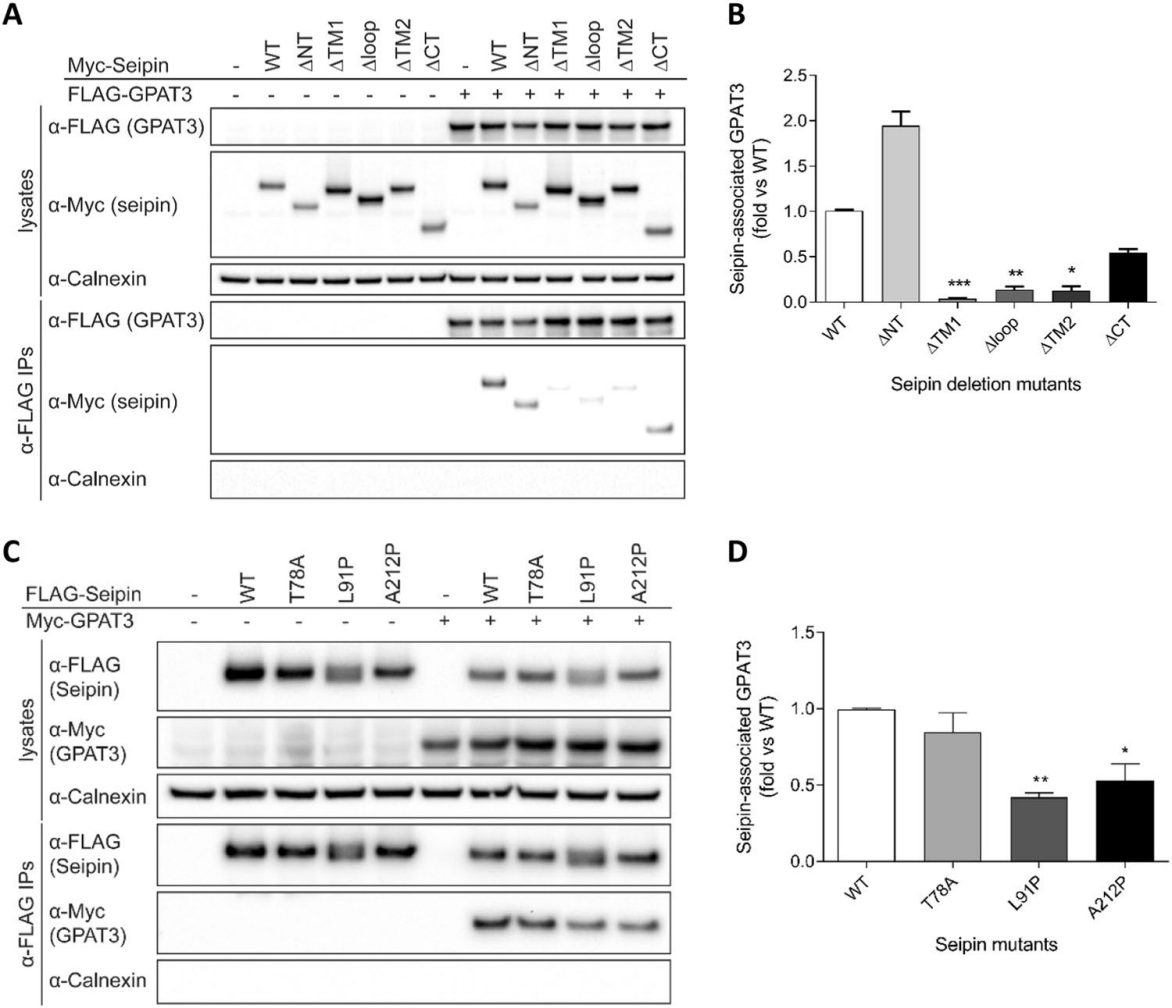
The conserved transmembrane and ER luminal loop regions of seipin are required for its interaction with GPAT3. (**A**) HEK293 cells were transfected with empty vector (−), wild-type Myc-seipin (WT) or mutant seipin lacking the N-terminus (ΔNT), the first transmembrane domain (ΔTM1), the ER luminal loop region (Δloop), the second transmembrane domain (ΔTM2) or the C-terminus (ΔCT) in the absence or presence of FLAG-tagged GPAT3 as indicated. Cell lysates or anti-FLAG immunoprecipitates were separated by SDS-PAGE and immunoblotted with antibodies to FLAG, Myc and calnexin. (**B**) Quantification analysis of the binding of WT or mutant forms of seipin to FLAG-GPAT3. Data represent means ± SEM (n = 3), normalized to expression levels and expressed as a fold of that observed with wild-type seipin. * indicates p < 0.05, ** indicates p < 0.01 and *** indicates p < 0.001 versus co-immunoprecipitation with wild-type seipin. (**C**) HEK293 cells were transfected with empty vector (−), N-terminal FLAG-tagged wild-type seipin (WT) or identically-tagged T78A, L91P and A212P forms of seipin in the presence or absence of Myc-tagged GPAT3. Cell lysates or anti-FLAG immunoprecipitates were separated by SDS-PAGE and immunoblotted with antibodies to FLAG, Myc and calnexin. (**D**) Quantification analysis of the binding of WT or mutant forms of seipin to Myc-GPAT3. Data represent the means ± SEM (n = 3), normalized to expression levels and expressed as a fold of that observed with wild-type seipin. * indicates p < 0.05, ** indicates p < 0.01 versus co-immunoprecipitation with wild-type seipin.

In order to examine the domains of seipin relevant for the interaction with GPAT3 we used deletion mutants of seipin lacking either the cytoplasmic N (ΔNT) or C termini (ΔCT), the first (ΔTM1) or second (ΔTM2) transmembrane domains or the ER luminal loop region (Δloop) of the protein (Fig. 2A,B). While deletion of either N or C termini of seipin did not significantly inhibit its interaction of with GPAT3, loss of either the first or second transmembrane domain or the ER luminal loop region of seipin significantly reduced GPAT3 binding. This finding contrasts with the interaction between seipin and AGPAT2, which does not require an intact second transmembrane domain^18^, indicating that the interaction of these two proteins with seipin occurs independently of one another.

In order to examine whether altered interaction with GPAT3 might contribute to the failure of adipose development in CGL2 patients we determined the capacity of GPAT3 to bind to pathogenic mutants of seipin bearing one of three single amino acid substitutions, T78A, L91P or A212P. All three mutant forms of seipin were able to co-immunoprecipitate GPAT3 (Fig. 2C). However, while the T78A mutant form of seipin was indistinguishable in this regard from wild-type seipin, the interaction of GPAT3 with L91P and A212P mutant forms of seipin was reduced by approximately 50% (Fig. 2C,D).

### GPAT3 binds directly to oligomers of seipin

**Figure 3:**
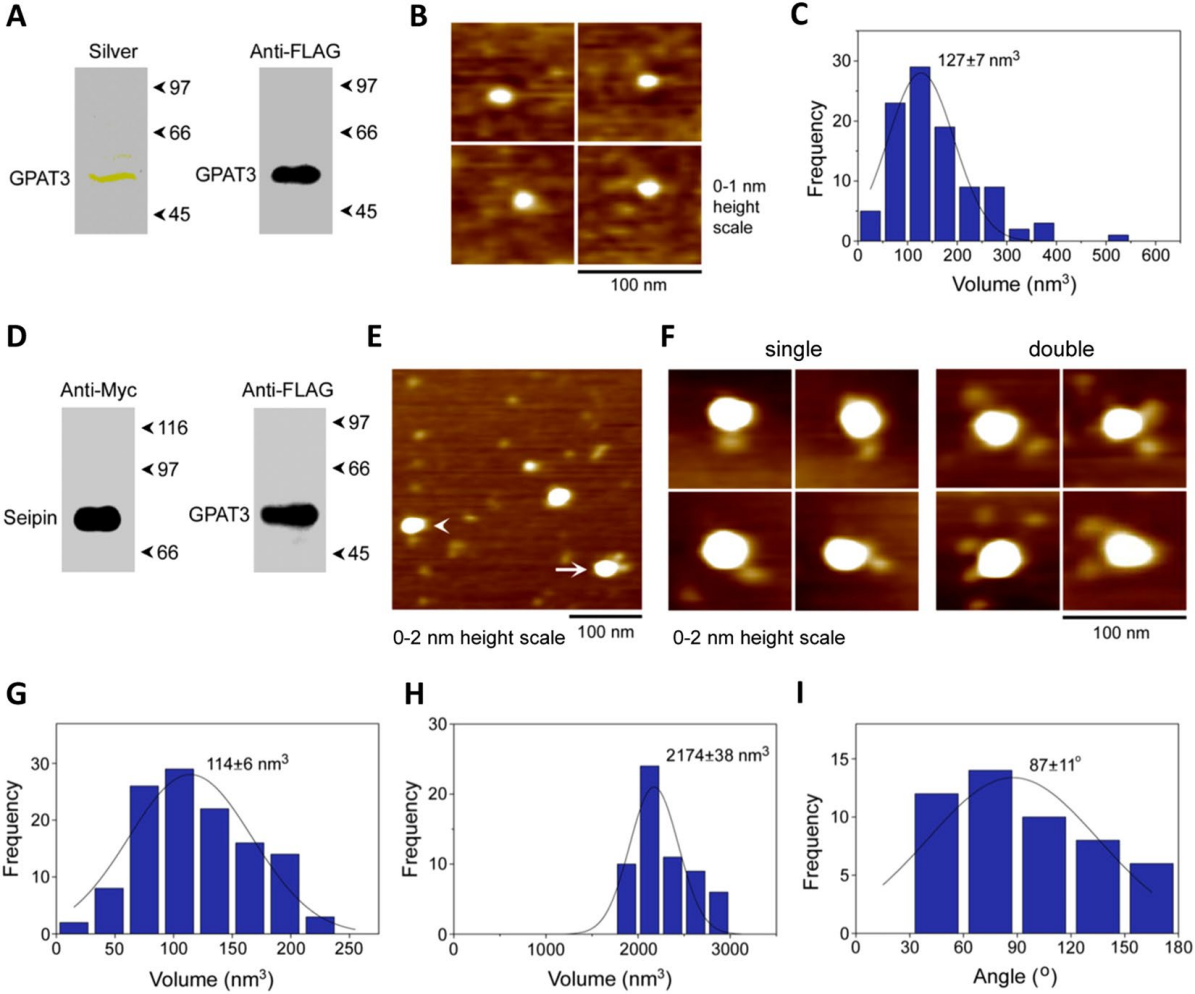
Seipin interacts with GPAT3 via direct physical contacts. (**A**) FLAG-GPAT3 was expressed in tsA 201 cells and isolated using anti-FLAG beads. Following SDS-PAGE, isolated protein was analysed by either silver staining (left panel) or immunoblotting using an anti-FLAG antibody (right panel). (**B**) Zoomed images from AFM analysis of immunoisolated GPAT3 showing individual GPAT3 particles. (**C**) Frequency distribution of volumes of the GPAT3 particles. The curve indicates the fitted Gaussian function. The peak of the distribution (±SEM) is indicated. (**D**) tsA 201 cells were co-transfected with FLAG-GPAT3 and Seipin-Myc and proteins were isolated using anti-Myc beads. Proteins were separated by SDS-PAGE followed by immunoblotting using either anti-Myc (left panel) or anti-FLAG (right panel) antibodies. (**E**) Low-magnification image from AFM analysis of isolated proteins. The arrowhead indicates a large particle (seipin) decorated by one smaller (GPAT3) particle; the arrow indicates a seipin particle decorated by two GPAT3 particles. (**F**) Gallery of zoomed images showing seipin particles decorated by either one (left panels) or two (right panels) GPAT3 particles. (**G**) Frequency distribution of volumes of the smaller (GPAT3) particles with the curve indicating the fitted Gaussian function. The peak of the distribution (±SEM) is indicated. (**H**) Frequency distribution of volumes of the larger (seipin) particles. (**I**) Frequency distribution of angles between pairs of bound GPAT3 particles.

We next investigated the molecular architecture of seipin/GPAT3 complexes using AFM. FLAG-GPAT3 was expressed in HEK-derived tsA 201 cells and immunoisolated (Fig. 3A), eluted and imaged by AFM (Fig. 3B). Analysis of the volumes of GPAT3 particles revealed a single peak at 127 ± 7 (mean ± SEM) nm^3^ (n = 100) (Fig. 3C), similar to the expected molecular volume of 104 nm^3^. FLAG-GPAT3 and seipin-Myc were then co-expressed and both proteins were subsequently detected in anti-Myc immunoprecipitates (Fig. 3D). Following elution of the co-immunoprecipitated proteins, AFM analysis revealed large particles with smaller, peripherally associated particles (Fig. 3E). Higher magnification images of these complexes are shown in Fig. 3F. The peak molecular volume of the peripheral particles (114 ± 6 nm^3^; n = 120; Fig. 3G) was very similar to that of GPAT3 alone (Fig. 3C). The peak volume of the core of the complex was 2174 ± 38 nm^3^ (n = 60; Fig. 3H). This is very similar to the volume of 2394 nm^3^ that we have reported for seipin dodecamers^12^. However, we note that human seipin has recently been reported to form 11-mers which could also be consistent with this molecular volume. Analysis of AFM images revealed that 10.4% (50/480) of seipin particles were decorated by two GPAT3 particles, although complexes with one GPAT3 particle were more common. In contrast, no complexes bearing peripherally associated particles were seen when seipin was expressed alone (158 particles). We also analysed the distribution of angles between pairs of bound GPAT3 molecules, which showed a peak at 87° ± 11° (n = 50; Fig. 3I). A toroidal, dodecameric arrangement of seipin oligomers should lead to an angle of 30° separation between individual seipin subunits^12^, whilst an 11-mer of seipin would result in an angle of 32.7° separation between subunits. Thus, our data imply that where two GPAT3 molecules bind to seipin subunits, these are typically separated by two unoccupied seipin subunits. Together, these AFM analyses show that oligomers of seipin can directly bind GPAT3, while the defined molecular architecture of these complexes indicates a highly ordered, specific interaction.

### Inhibition of GPAT3 expression is unable to rescue seipin deficiency in differentiating adipocytes and GPAT3 overexpression does not impair adipogenesis

**Figure 4:**
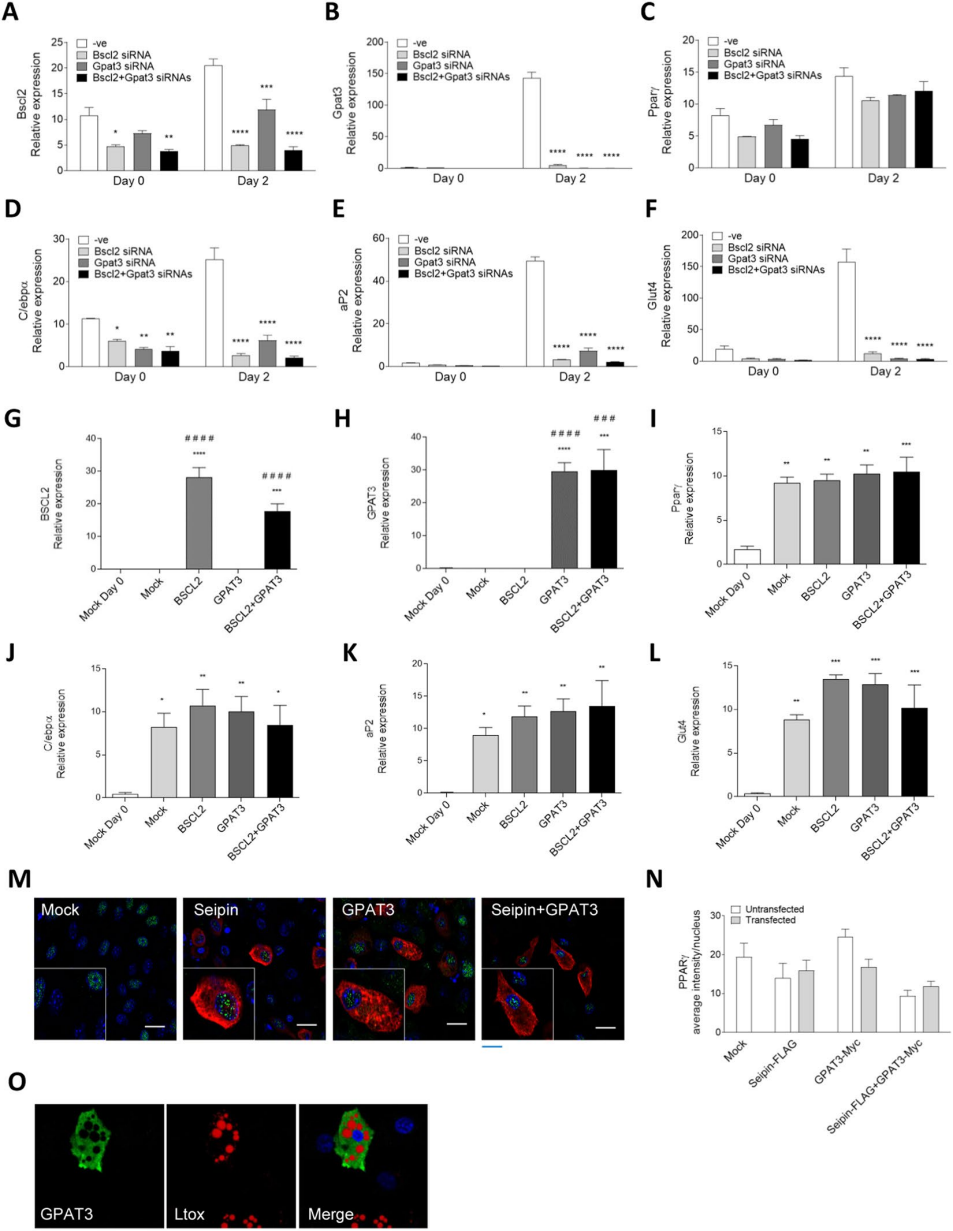
Inhibition of Gpat3 expression fails to rescue adipogenesis in seipin-deficient cells, while GPAT3 overexpression does not impair this process. C3H10T1/2 cells were transfected with control siRNA (−ve) or siRNA targeting seipin (Bscl2), Gpat3 or both seipin (Bscl2) and Gpat3 as indicated at day -2 and day 0 of differentiation. RNA was extracted at day 0 or day 2 of differentiation and expression of Bscl2/seipin (**A**) Gpat3 (**B**) Pparγ (**C**) C/ebpα (**D**) aP2 (**E**) and Glut4 (**F**) was determined by qPCR. Data are presented as relative mRNA expression (means ± SEM, n = 4), normalized to the stable housekeeping gene Ywhaz. Statistically significant differences compared to control siRNA at each time point are indicated: * indicates p < 0.5, **p < 0.01, ***p < 0.001, ****p < 0.0001. Alternatively, cells were transfected at day -2 and day 0 of differentiation with empty vector or plasmids encoding seipin (BSCL2), GPAT3, or co-transfected with both seipin (BSCL2) and GPAT3, as indicated. RNA was isolated at day 0 or day 2 of differentiation and expression of BSCL2/seipin (**G**), GPAT3 (**H**), Pparγ (**I**), C/ebpα (**J**), aP2 (**K**) and Glut4 (**L**) determined by qPCR. Data are presented as means ± SEM, (n = 4), normalized to Ywhaz. Statistical analysis was made by one-way ANOVA followed by Dunnett’s multiple comparison post-hoc test. Statistically significant differences compared to control day 0 (Mock Day 0) are indicated by *p < 0.05, **p < 0.01, ***p < 0.001, ****p < 0.0001. Statistically significant differences compared to control (Mock day 2) samples are indicated by ^###^p < 0.001, ^####^p < 0.0001. (**M**) Identically transfected cells were also differentiated for 3 days, fixed and immunostained to detect FLAG-seipin/BSCL2 (red) or Myc-GPAT3 (red), anti-PPARγ (green) and DAPI for nuclei (blue). Co-transfected cells were immunostained for myc-GPAT3. (**N**) Quantified PPARγ signal intensity in the nuclei of untransfected and transfected cells in 50 nuclei per group in two independent experiments. Scale bar, 20 µm. Data represent means ± SEM. (**O**) Representative image of C3H10T1/2 overexpressing GPAT3 fixed at day 5 of differentiation and immunostained with antibodies to Myc-GPAT3 (green) and LipidTox to visualize lipid droplets.

The previous report of reduced adipogenesis in cells lacking GPAT3^20^, and our own data from preadipocytes in which GPAT3 was inhibited (Fig. S1A–D), appeared to directly contradict the more recent report that seipin may exert its proadipogenic effects by inhibiting GPAT3^19^. Although they did not examine the effect of inhibiting GPAT3 on adipogenesis, the authors of the latter study did report that inhibition of GPAT3 expression could rescue adipogenesis in cells lacking seipin^19^. This led them to propose that inhibiting GPAT3 could offer a potential therapy for CGL2. We decided to examine this using knockdown of seipin (Bscl2) and/or Gpat3 by siRNA in C3H10T1/2 cells. Transfection with specific siRNA two days prior to the induction of differentiation efficiently and selectively targeted either seipin (Fig. 4A) or Gpat3 (Fig. 4B) mRNA expression at day 0 when adipogenesis was induced. This was the case whether used alone or in combination. As shown previously in Fig S1B, our Gpat3-targetting siRNA does not affect the expression of Gpat4 mRNA prior to adipogenic induction. Inhibition of seipin or Gpat3 did not significantly affect Pparγ induction at this early stage of differentiation, but did lead to significant inhibition of C/ebpα, aP2 and Glut-4 expression during early adipogenesis (Fig. 4C–F). When both Gpat3 and seipin were inhibited, this led to an even more marked reduction in the induction of these key markers of adipocyte differentiation. To determine whether this effect was sustained we also examined lipid accumulation in C3H10T1/2 cells in which seipin and/or Gpat3 expression had been inhibited with siRNA followed by differentiation for 10 days. Inhibition of seipin expression significantly reduced total lipid accumulation in these cells (Fig. S2A,B). In contrast, lipid accumulation in cells in which Gpat3 expression had been inhibited was indistinguishable from that in control cells. However, Gpat3 expression failed to rescue lipid accumulation in cells lacking seipin. Hence, whilst the initial impairment of adipogenic gene induction caused by Gpat3 inhibition did not ultimately cause a significant reduction in lipid accumulation, we found no evidence that additional loss of Gpat3 could rescue differentiation caused by the loss of seipin. As these data are at variance with the observations in the report from Pagac *et al*. we also performed experiments in the 3T3-L1 preadipocyte cell line used in their study^19^. This produced similar effects whereby siRNA-mediated inhibition of either seipin or Gpat3 (Fig. S3A,B) led to inhibition of the induction of the adipogenic markers Pparγ, C/ebpα, aP2 and Glut-4 (Fig. S3C–F). With the exception of Glut-4, additional inhibition of Gpat3 exacerbated the inhibition of adipogenesis by seipin loss during early adipogenesis. We also repeated these experiments with an additional siRNA targeting seipin alone or in combination with two different siRNA targeting Gpat3. These alternative siRNA were not as effective at inhibiting seipin or Gpat3 expression (Fig. S3G,H). Nonetheless, we observed similar results to those obtained with the original combination of siRNA when the early induction of the adipogenic markers Pparγ, C/ebpα, aP2 and Glut-4 were examined following two days of differentiation (Fig. S2G–L). Where the effect of combined knockdown differed from that of seipin or Gpat3 inhibition alone, this was to further impair the induction of these genes. We also verified that the second Gpat3 siRNA did not directly inhibit Gpat4 mRNA expression (Fig. S3M).

To further test whether GPAT3 might act as an inhibitor of adipogenesis we next determined the effect of overexpression of GPAT3 and/or seipin on adipocyte differentiation. Transfection of C3H10T1/2 cells with either FLAG-Seipin or Myc-GPAT3 prior to induction of differentiation led to their robust expression (Fig. 4G,H). When markers of adipogenesis were examined at day 3 of differentiation in these cells we observed significant induction of Pparγ, C/ebpα, aP2 and Glut-4 mRNA expression regardless of whether seipin, GPAT3 or both proteins were overexpressed (Fig. 4I–L). Critically we saw no evidence that GPAT3 overexpression inhibited adipogenesis; rather this appeared to modestly enhance the process. As these data contradicted those of Pagac *et al*., we considered whether our use of transient transfection could mean that cells expressing GPAT3 have reduced adipogenesis but that this effect might be masked by untransfected cells in the cultures. Hence, we transfected cells with either FLAG-seipin or Myc-GPAT3 alone or in combination and then individually analysed the accumulation of PPARγ in transfected versus untransfected cells in each culture, as a marker of adipogenic induction. In cultures of cells transfected with either FLAG-seipin, Myc-GPAT3 or both proteins, we observed no significant reduction in the overall fluorescence intensity of nuclear PPARγ staining between transfected and untransfected cells with any of the transfection conditions examined (Fig. 4M,N). Moreover, we transfected preadipocytes with GPAT3 before the induction of adipogenesis and then differentiated the cells to mature adipocytes. In agreement with our data analysing nuclear PPARγ accumulation during early adipogenesis, the absence or presence of GPAT3 expression in individual cells had no detectable effect on the formation of mature adipocytes as assessed by the appearance of lipid droplets (Fig. 4O). Finally, having noted that GPAT3 overexpression may modestly increase the induction of adipogenic markers in C3H10T1/2 cells (Fig. 4I–L), we also examined the effect of GPAT3 overexpression in 3T3-L1 cells, the line used by Pagac *et al*.^19^. In 3T3-L1 cells transfected with human GPAT3, endogenous murine Gpat3 expression was unaffected but we observed significantly increased expression of Pparγ, C/ebpα, aP2, adiponectin and perilipin mRNA (Fig. S3N). Although we did not formally assay the specific activity of GPAT3 in these cells we believe these data strongly imply that the GPAT3 construct used in our studies is functionally active. Overall, we find no evidence that robust expression of GPAT3 leads to any inhibition of adipogenesis. Indeed our data suggests that GPAT3 expression may positively influence adipocyte development, consistent with the substantial induction of its expression during early adipogenesis.

### Seipin can bind both AGPAT2 and GPAT3, facilitating the interaction between these key adipogenic enzymes

**Figure 5:**
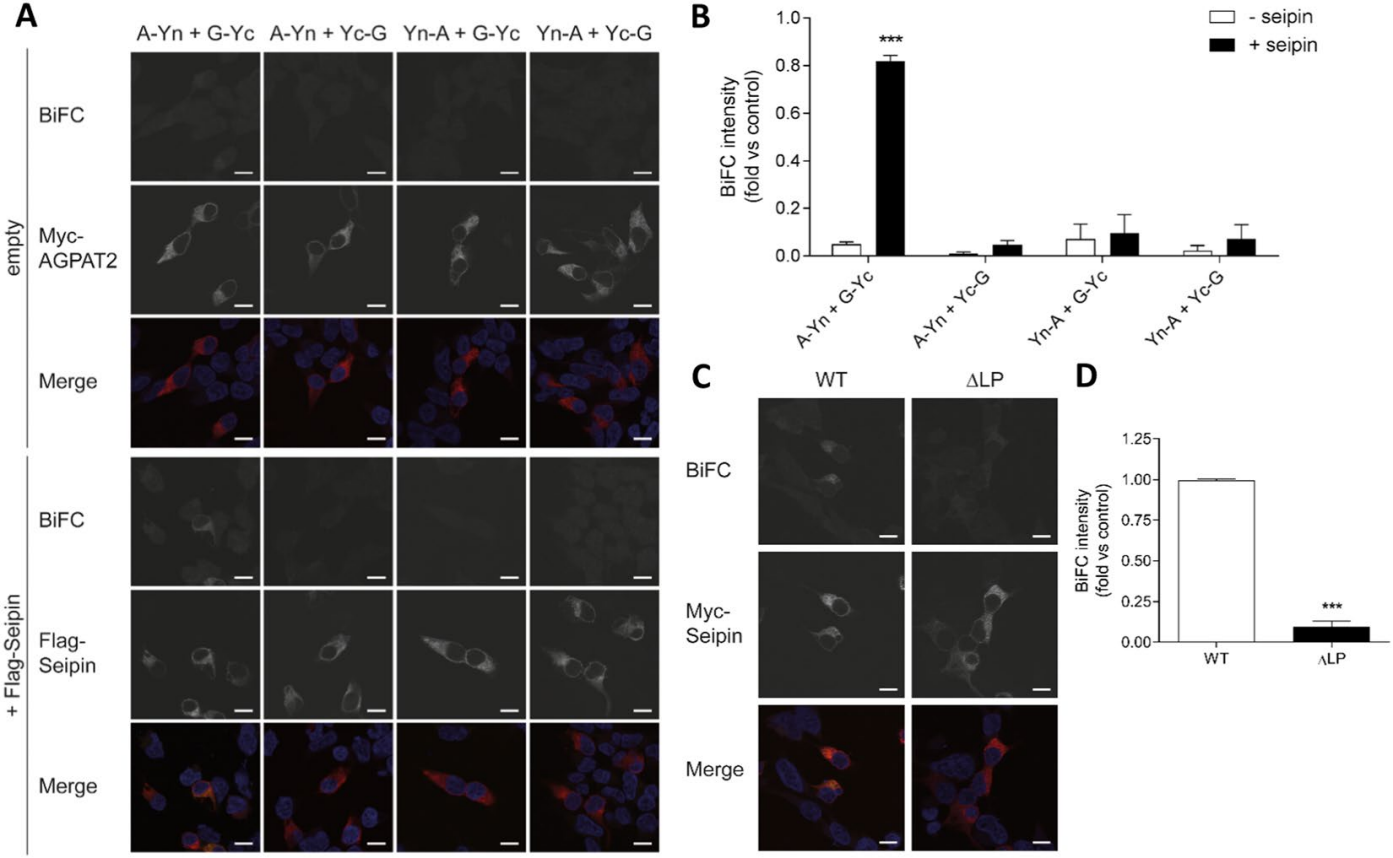
Seipin potentiates the interaction between AGPAT2 and GPAT3. (**A**) HEK293 cells were co-transfected with constructs in which the N terminus of YFP was fused to the N terminus (Yn-A) or C terminus (A-Yn) of AGPAT2-Myc, and with constructs in which the C-terminus of YFP was fused to the N terminus (Yc-G) or C terminus (G-Yc) of GPAT3, in the absence or presence of FLAG-tagged seipin. Following a temperature shift to induce the formation of reconstituted YFP, cells were fixed and immunostained for Myc (AGPAT2) or FLAG (seipin), and DAPI to label nuclei. The interaction between AGPAT2 and GPAT3 is indicated by the presence of a YFP signal. Individual images are shown in grayscale and merged images show overlay of YFP (yellow) and AGPAT2-Myc or FLAG-seipin (red). Scale bars, 10 μm. (**B**) Quantified total BiFC signal intensity per image in 20 random images each of 3 separate experiments, normalized against a BiFC positive control, where cells were transfected with GPAT3-Yc and seipin-Yn. Data represent means ± SEM (n = 3). *** indicates p < 0.01 versus co-immunoprecipitation in the absence of seipin. (**C**) HEK293 cells were co-transfected with AGPAT2-Yn and GPAT3-Yc constructs in the presence of wild-type Myc-tagged seipin or identically-tagged mutant seipin lacking the loop domain (ΔLP). Following a temperature shift to induce the formation of reconstituted YFP, cells were fixed and stained for Myc-seipin and DAPI to label nuclei. The indirect interaction between AGPAT2 and GPAT3 in the presence of seipin is indicated by the presence of YFP signal. Individual images are shown in grayscale, and merged images show overlay of YFP (yellow) and Myc-seipin (red). Scale bars, 10 μm. (**D**) Quantified total BiFC signal intensity per image in 20 random images, each of 3 separate experiments. Data shown represent the means ± SEM (n = 3). *** indicates p < 0.001 versus BiFC signal observed in the presence of wild-type seipin.

We have previously shown that seipin can bind AGPAT2, which uses the product of GPAT3 activity, lysophosphatidic acid, as a substrate. Next, we employed BiFC analysis to investigate whether seipin might act as a scaffold to co-ordinate the association of these two enzymes. To do this, the N-terminal portion of YFP (Yn) was fused to the N or C terminus of AGPAT2 to generate Yn-AGPAT2 and AGPAT2-Yn. Similarly, the C-terminal portion of YFP (Yc) was fused to either the N or C terminus of GPAT3 to generate Yc-GPAT3 or GPAT3-Yc, respectively. All combinations of these fusion proteins were then expressed in HEK293 cells in the absence or presence of co-expressed seipin. Reconstituted YFP fluorescent signal was clearly detectable in cells expressing AGPAT2-Yn and GPAT3-Yc, specifically when seipin was co-transfected with these constructs but not when seipin was absent (Fig. 5A,B). However, no BiFC signal was detected when any other combination of AGPAT and GPAT3 BiFC constructs was used. To further test the specificity of this scaffolding function we compared the capacity of wild-type seipin and the mutant form of seipin lacking the ER luminal loop domain of the protein (ΔLP) to induce BiFC fluorescence when co-expressed with AGPAT2-Yn and GPAT3-Yc. This mutant form of seipin was chosen as it retains the transmembrane domains and has been shown to have the same overall structure and localisation as the wild-type protein^23^ but has reduced binding to GPAT3 (Fig. 2A,B) and AGPAT2^18^. Despite equivalent expression of wild-type and ΔLP-seipin, the latter was not able to induce a BiFC signal between AGPAT2-Yn and GPAT3-Yc (Fig. 5C,D).

### Inhibition of either seipin, AGPAT2 or GPAT3 impairs adipogenesis and prevents the early induction of Akt activity

**Figure 6:**
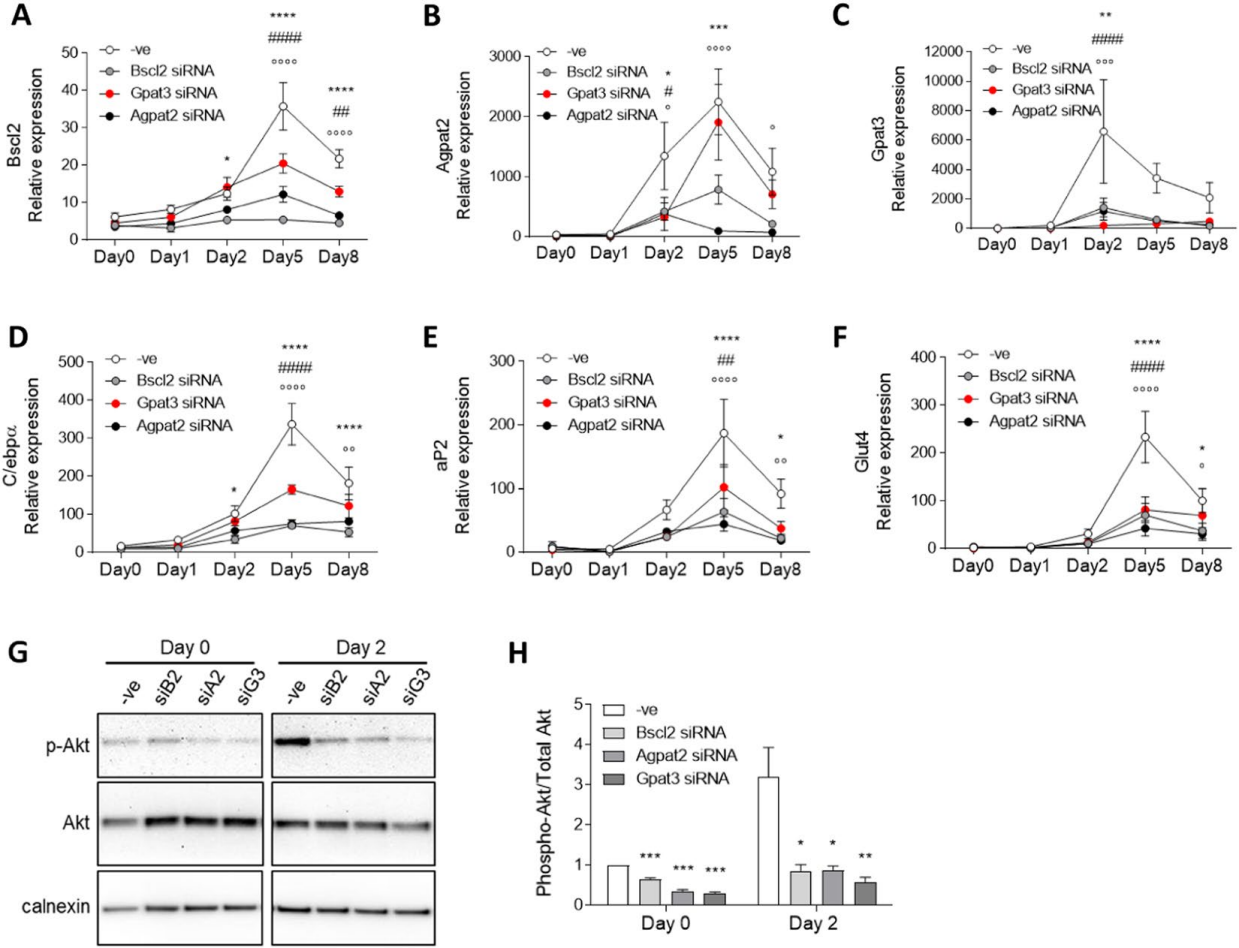
Inhibition of either seipin, Gpat3 or Agpat2 expression similarly inhibits adipogenesis. C3H10T1/2 cells were transfected with control siRNA (−ve) or siRNA targeting seipin (Bscl2), Agpat2 or Gpat3 as indicated at day -2 and day 0 of differentiation. RNA was extracted at various time points and expression of seipin (Bscl2) (**A**) Agpat2 (**B**) and Gpat3 (**C**) C/ebpα (**D**) aP2 (**E**) and Glut4 (**F**) was determined by qPCR. Data represent means ± SEM (n = 4), normalized to Ywhaz. Statistical analysis was made with one-way ANOVA followed by Dunnett’s multiple comparison test. At each time point, significant differences between Bscl2 siRNA samples *versus* control siRNA samples are indicated by *p < 0.05, **p < 0.01, ***p < 0.001, ****p < 0.0001; between Gpat3 siRNA samples *versus* control siRNA samples by ^#^p < 0.05, ^##^p < 0.01, ^####^p < 0.0001; and between Agpat2 siRNA samples *versus* control siRNA samples by °p < 0.05, °°p < 0.01, °°°p < 0.001, °°°°p < 0.0001. (**G**) Identically-transfected cells were lysed and analysed for total and phospho-Akt (Ser-473) levels by immunoblotting at either day 0 or day 2 of differentiation as indicated. Calnexin was used as a loading control. Full blots are shown in Fig. S4. (**H**) Quantification of the ratio of phosho-Akt to total Akt protein. Data represent means ± SEM (n = 3); expression is relative to total Akt levels in the same samples. Statistically significant differences from expression in control siRNA transfected cells (-ve) are indicated by *p < 0.05, **p < 0.01, ***p < 0.001.

As our data indicated that seipin, GPAT3 and AGPAT2 might act together in a co-ordinated manner, we directly compared the effects of inhibiting these three proteins in differentiating C3H10T1/2 cells. Inhibition of either seipin, Gpat3 or Agpat2 using siRNA (Fig. 6A–C) led to a significant reduction in the expression of the key adipogenic markers C/ebpα, aP2 and Glut-4 (Fig. 6D–F). It has previously been reported that loss of Akt activation during early adipogenesis may underpin the failure of adipocyte development in Agpat2 deficient preadipocytes^24^. We therefore examined whether this may be a common feature of seipin, Agpat2 and Gpat3 deficiency. While phosphorylation of Akt was significantly increased in C3H10T1/2 cells at day 2 of adipogenesis, inhibition of Agpat2 expression prevented this (Fig. 6G,H), consistent with the previous findings of Subauste *et al*.^24^. Inhibition of either seipin or Gpat3 expression also completely prevented the induction of Akt phosphorylation in these cells. Overall, these data are consistent with a model in which seipin, GPAT3 and AGPAT2 act together in a co-ordinated manner to promote adipogenesis in the early stages of this process.

## Discussion

In this study, we have confirmed a previous report that the protein seipin, which plays a critical role in adipose tissue development and maintenance in humans, can bind to the glycerol-3 phosphate acyltransferase enzymes GPAT3 and GPAT4^19^. In addition, our data suggest that GPAT3 may be the more important of the GPATs during early adipogenesis, consistent with previous studies^19,20^. Hence, we focussed on the interaction between GPAT3 and seipin, and whether the loss of this association could mediate some of the effects of seipin deficiency on adipogenesis. Extending the studies of Pagac *et al*., we further define the regions of seipin required for the interaction with GPAT3 and show that the pathogenic A212P and L91P mutant forms of seipin have a reduced capacity to bind to GPAT3. We have previously reported that these two mutant forms of seipin are unable to appropriately form uniform oligomers in the same way as the wild-type protein, that they partially mislocalise to the nuclear envelope, and that they have modestly but significantly decreased expression in preadipocytes^8,12^. A combination of these factors, along with reduced binding to GPAT3, may explain the inability of these mutant forms to support adipose development in affected patients in the same way as wild-type seipin.

We were also able to demonstrate, using AFM analysis, that the interaction of GPAT3 with seipin involves direct contact between the two proteins. These data revealed that multiple molecules of GPAT3 could bind to a single seipin oligomer. The distribution of GPAT3 molecules bound to a single seipin oligomer was not random, further demonstrating that the association between these two proteins is specific. Unfortunately, we were unable to use AFM to analyse whether single seipin homooligomers can bind to both GPAT3 and AGPAT2, as we have previously done for AGPAT2 and lipin 1^18^. This experiment would require a significant difference in size between the proteins being analysed for them to be unequivocally distinguished from each other by AFM, and GPAT3 and AGPAT2 do not differ sufficiently in their molecular volumes. However, our BiFC analyses show that GPAT3 and AGPAT2 are extremely closely juxtaposed, selectively when seipin is present, indicating that they can indeed both bind to a single seipin oligomer.

In contrast to the findings of Pagac *et al*.^19^, we did not find that inhibiting GPAT3 could rescue adipogenesis in seipin-deficient cells. Indeed, additional loss of GPAT3 expression exacerbated the failure of adipocyte differentiation in cultured cells under these conditions. We were also unable to detect any impairment of adipogenesis in preadipocytes overexpressing GPAT3, even when assessed at a single cell level. Indeed, overexpression of GPAT3 modestly potentiated the induction of adipocyte genes in 3T3-L1 preadipocytes (Fig. S3N). The reason for this inconsistency is not clear but may relate to the levels of overexpression of GPAT3 in each study. Alternatively, we note that Pagac *et al*., used stably transduced cells and it is possible that the effects of long-term expression of GPAT3 in preadipocytes differs from the acute expression achieved in our model by transient transfection two days prior to adipogenesis. In a similar manner, we acutely inhibited seipin and/or GPAT3 in our double knockdown experiments, whilst Pagac *et al*., used stably transduced cells. Again, the latter may lead to compensatory changes in the preadipocytes or effects that only manifest with sustained suppression of these genes during cell proliferation prior to the induction of adipogenesis. These may not be replicated in our experimental model by the acute inhibition of these genes in confluent cells immediately preceding adipogenesis. Whilst less likely in our view, it is also possible that differences in the levels of suppression achieved by each shRNA/siRNA in the different studies, or even off-target effects, could have led to the apparent discrepancies observed between the findings of Pagac *et al*., *versus* our data and the earlier work of Shan *et al*.^20^.

Our experiments revealed that seipin could facilitate the association of GPAT3 with AGPAT2, which can utilise the product of GPAT3 in the same glycerolipid synthesis pathway. AGPAT2 is required for adipogenesis and its disruption, like that of seipin, causes CGL in affected patients. Loss of seipin, GPAT3 or AGPAT2 impaired adipogenesis in our analyses, although the effect of inhibiting GPAT3 was clearly less dramatic than the loss of either AGPAT2 or seipin. Overall, these results lead us to conclude that seipin may not act as an inhibitor of GPAT3 activity in the context of early adipogenesis. Rather, we speculate that seipin might potentiate adipocyte differentiation by scaffolding together the acyltransferases GPAT3 and AGPAT2 and the PA phosphatases lipin 1 and/or 3, all of which positively regulate adipogenesis. This need not involve any alteration of the specific activity of each enzyme but rather facilitate co-localisation of these proteins, possibly permitting the efficient delivery of the product of each enzyme as substrates for the next within this single pathway.

In their study Pagac *et al*., used a variety of carefully performed and detailed enzymatic assays to demonstrate that seipin overexpression may lead to reduced GPAT activity in a variety of cell types^19^. Our findings do not necessarily contradict these results as, particularly in cells other than differentiating preadipocytes, there may be circumstances in which seipin performs this function. For example, seipin may sequester GPAT3 to a specific microdomain of the ER, reducing GPAT3 activity at the surface of lipid droplets. Similarly, seipin may impair the access of exogenous substrate to GPAT enzymes in these assays, leading to reduced detectable GPAT3 specific activity. Interestingly, it has been reported recently that elevated GPAT3 activity can mediate increased cellular stress in conditions of lipotoxicity^25^. This study examined the effect of exogenous lipid on non-adipose cells but clearly demonstrates the potential of increased GPAT3 activity to cause deleterious effects to cell function in some cell types. It will be interesting to determine whether seipin could alter the ability of lipids to induce cellular stress via GPAT3 inhibition in this context.

Our data, as those of Shan *et al*.^20^, show that inhibition of GPAT3 can impair the induction of some adipocyte markers during the early stages of differentiation in cellular models of this process. However, we find that GPAT3 inhibition leads to a more modest impairment of adipogenesis than seipin or AGPAT2 loss, so that lipid accumulation and the expression of some adipogenenic marker genes such as C/ebpα and Glut4 are not significantly reduced when analysed at later stages of adipogenesis (see Fig. S2A,B and Fig. 6D,E). Of note, GPAT3-null mice have apparently normal fat development, which is consistent with GPAT3 being less critical than seipin or AGPAT2 in the development of mature adipocytes and may even be entirely dispensable *in vivo*^21^. However, female, but not male, GPAT3-null mice have reduced adipose tissue expansion following high fat feeding which could result from a modestly reduced capacity to expand adipose tissues^21^. Whilst this manuscript was in revision, Gao and colleagues reported that additional disruption of GPAT3 can improve insulin tolerance and reduce hepatic steatosis in seipin-null mice^26^. Like seipin-null mice, mice lacking both GPAT3 and seipin exhibit a very striking reduction in white adipose tissue mass compared with control mice. However, BAT mass is increased in GPAT3/seipin double knockout mice and residual white adipose depots showed modestly increased mass and increased browning^26^. Thus, additional loss of GPAT3 appears to increase BAT development and improves metabolic health without substantially rescuing white adipogenesis in this model of CGL2.

GPAT4-null mice show reduced body weight, increased energy expenditure and reduced adipose expansion in response to a high fat diet with a notable loss of subdermal adipose tissue^27^. This may particularly reflect an important role for GPAT4 in lipid storage *versus* oxidation in brown adipose tissue where it is highly expressed^27,28^. The complexity of these cellular and *in-vivo* phenotypes may result from differential roles of each isoform in regulating adipogenesis in different adipose depots in combination with additional roles in triglyceride synthesis and lipid oxidation. More targeted models of GPAT disruption *in vivo* will be required to clarify how findings from cultured cells, including those presented here, translate to their function in both murine and human adipose tissue *in vivo*. AGPAT2 deficiency leads to the loss of nearly all adipose depots in humans but so-called “mechanical” depots are spared^29^. However, while all WAT depots are absent in seipin-deficient CGL patients, including those spared in AGPAT2 deficiency, seipin appears dispensable for BAT development, at least in mice^7,30,31^. Thus, it is increasingly apparent that the pathways governing the development and expansion of adipose mass differs significantly between depots depending on location and the identity of the stem cells involved^32,33^. Such complexity is also likely to apply when considering the roles of different GPAT isoforms in these processes.

Several studies have examined the evolutionarily conserved role of seipin in lipid droplet biogenesis. GPAT3 and GPAT4 have important roles in lipid droplet growth in multiple cells, including adipocytes. In particular, GPAT4 has been shown to accumulate on lipid droplets during droplet expansion^34^. Seipin has been shown to partly localize to ER-LD contact sites, and so may be involved in recruitment of GPAT and other lipogenic enzymes to this location to alter lipid droplet biogenesis, growth or budding^14,35^. In addition to GPATs, AGPAT2 and lipins, a range of other proteins have been identified as seipin interacting partners that may regulate lipid droplets in multiple cell types and species. These include ADRP, SERCA2A, 14-3-3β, perilipin, promethin and Reep1 in mammalian cells^17–19,36–41^ and Pet10p, Ldo45 and Ldo16 in yeast^42–44^. In addition, two groups have recently reported the structure of seipin homo-oligomers determined by cryo-EM. The first examined the Drosophila form of the protein and revealed a 12-subunit complex^13^. The authors proposed an elegant model whereby these may scan the ER for nascent LD structures. Moreover, seipin oligomers may have an unusual capacity to switch conformation to adapt to the membrane structures that occur at these ER-LD interfaces. In this way they may act principally as physical controllers to regulate droplet dynamics. Alternatively, seipin may be able to perform this scanning role but also recruit appropriate binding partners to influence droplet size or composition. A second study using cryo-EM has reported that human seipin forms 11-subunit oligomers^15^. Our own studies using AFM suggest 12 subunits^12^. However, both the distribution of molecular volumes and the distributions of angles we observed between antibodies bound to seipin homo-oligomers could allow for either 11-mers or 12-mers^12^. The differences between our findings by AFM and cryo-EM analysis of human seipin might also reflect the expression and isolation methods used in each case. Yan *et al*. also showed that seipin may act as a phospholipid binding protein^15^. This could influence membrane curvature and so contribute to the ability of seipin to affect LD dynamics. It is not clear whether the evolutionarily conserved role of seipin in the regulation of lipid droplets is related to or distinct from its critical role in adipocyte differentiation. It seems credible that seipin’s capacity to structurally adapt to LDs and its interaction with LD proteins do not directly affect the process of adipogenesis. Meanwhile, the interaction with enzymes important for adipogenesis and LD function, such as GPATs, AGPAT2 and lipins may underlie the requirement for seipin in adipogenesis or have relevance to both functions.

Overall, our data confirm the previous report that seipin can bind to GPAT3. We reveal that this interaction involves direct association between the two proteins and present evidence that seipin oligomers may scaffold together both AGPAT2 and GPAT3 in a single multi-protein complex. However, we find no evidence that the loss of seipin causes a failure of adipogenesis due to increased GPAT3 activity. Rather our data support earlier work indicating that GPAT3 activity positively regulates adipocyte differentiation. Together, we believe that our work supports a model whereby seipin oligomers act as a scaffold for AGPAT2 and GPAT3, co-ordinating their proadipogenic activities. Importantly, they also suggest that, whilst there is recent evidence that inhibiting GPAT3 might improve some metabolic parameters in *BSCL2* deficiency, this is unlikely to substantially increase white adipose tissue mass in CGL2 patients.

## Materials and Methods

### Cell culture

HEK293 cells were grown in DMEM containing 10% FBS and transiently transfected using calcium phosphate precipitation or Fugene 6 transfection reagent (Promega) as in^12^. C3H10T1/2 cells and 3T3-L1 preadipocytes were maintained and differentiated as described previously^17^. Differentiating adipocytes were transiently transfected as in^17^ using lipofectamine LTX (Invitrogen). Transient knockdown in 3T3-L1 or C3H10T1/2 cells was carried out using Lipofectamine RNAiMAX transfection reagent (Life Technologies), two days prior to and at the induction of adipogenesis, following the manufacturer’s protocol. siRNAs were from ThermoFisher Scientific (with catalogue numbers) as follows: Control siRNA (4390844), Bscl2 #1 (s66847), Bscl2 #2 (s66848), Gpat3 #1 (s106867), Gpat3 #2 (s106865), Agpat2 (s85202). For BiFC experiments, 3T3-L1 or HEK293 cells were cultured on glass coverslips, and transfected with S-Yn, Yn-S, G-Yc and Yc-G plasmids using Lipofectamine LTX (Invitrogen) or Fugene 6 (Roche), respectively. Cells were maintained at 37 °C for 4 h, then at 32 °C for 20 h and then at 30 °C for 2 h. Cells were harvested or fixed, permeabilized, blocked and analysed as previously described^17^. For AFM experiments, tsA 201 cells were cultured in DMEM supplemented with 10% FBS and transfected using calcium phosphate precipitation. Cells in 5 × 162 cm^2^ culture flasks were transfected with 250 μg of DNA, and cells were harvested after 48 h and used to immunoprecipitate proteins for AFM analysis.

### Constructs

Constructs to express FLAG-seipin, FLAG-AGPAT2, FLAG-GPAT3 and FLAG-seipin-Myc were generated in the pCMV3xFLAG vector (Sigma Aldrich). Seipin-Myc, GPAT3-Myc, GPAT4-Myc and AGPAT2-Myc were in the pcDNA3.1MycHis vector (LifeTechnologies), as previously described^8,12,17,45^. Fusion constructs for BiFC experiments were generated essentially as in^17^. Briefly, the N-terminal (1–158) fragment of YFP was inserted downstream or upstream of seipin in pCMV3xFLAG to generate S-Yn and Yn-S fusion constructs, respectively. Similarly, the N-terminal fragment of YFP was inserted upstream or downstream of AGPAT2 or AGPAT2-Myc to generate tagged and untagged forms of A-Yn and Yn-A fusion proteins. The C-terminal (155–239) fragment was amplified and inserted downstream or upstream of GPAT3 or GPAT3-Myc to generate G-Yc and Yc-G fusion constructs, respectively.

### Immunoprecipitations and immunoblotting

Lysates and anti-FLAG or anti-Myc immunoprecipitates were prepared as described in^12,17^. HEK293 cells were transfected and 48 h later lysed in n-octyl-β-D-glucopyranoside (ODG) lysis buffer comprising 50 mM ODG, 50 mM Tris, pH 6.8, 150 mM NaCl, 1 mM EDTA with protease inhibitors (Complete EDTA-free, Roche Applied Science) and phosphatase inhibitor cocktails (Sigma). Cells were sonicated for two cycles of 30 s at medium intensity, incubated for 20 min on ice, centrifuged at 16,000 g for 10 min at 4 °C and supernatants removed for use. Lysate containing 1 mg of protein was incubated with 30 μl of anti-FLAG-agarose beads (Sigma-Aldrich) which had been pre-equilibrated with ODG lysis buffer in a total volume of 800 μl. Following rotation for 2 h at 4 °C, samples were centrifuged (8,200 g for 30 s at 4 °C), supernatants were removed and beads washed three times with ODG lysis buffer and any excess buffer removed. FLAG-tagged proteins were recovered from beads using 200 ng/μl 3×FLAG peptide (Sigma-Aldrich) in TBS (50 mM Tris–HCl, pH 7.4, 150 mM NaCl) and incubated at 4 °C with rotation for 30 min. After centrifugation supernatants were transferred to new tubes. 20 μg of lysate and 20 μl of IP samples were incubated with lithium dodecyl sulfate (LDS) sample buffer (Invitrogen). Samples were heated to 95 °C for 5 min, except those to be probed for seipin, and then separated by SDS-PAGE and transferred onto nitrocellulose membranes. Immunoblots were probed with antibodies to FLAG (Sigma), Myc (clone 4A6 Millipore), Akt, Phospho-Akt (Ser-473) (Cell Signaling) or calnexin (Abcam), followed by horseradish peroxidase (HRP)-linked secondary antibodies (Cell Signaling & Thermo Scientific) and visualized by ECL.

### BiFC, immunofluorescence and lipid staining

Antibodies used in immunofluorescence analyses were as described above for immunoblotting. Highly cross-absorbed Alexa Fluor anti-mouse 488, anti-mouse 594 or anti-rabbit 594 secondary antibodies were used for detection (Invitrogen). Image acquisition and quantification of BiFC signals were as described in^17^. For Oil red O analysis of neutral lipid accumulation in adipocytes, following fixation, cells were treated with Oil Red O 0.1% solution further diluted in dH2O (6 parts Oil Red O 0.1% in 4 parts dH2O). Images were taken at Olympus IX-50 inverted microscope, 10 × magnification. B). Neutral lipid content was then quantified following spectrophotometric reading at 520 nm with SpectraMax 190 (Molecular Devices).

### AFM imaging of isolated proteins

tsA 201 cells were transfected, lysed and proteins isolated by immunoprecipitation using anti-Myc, anti-HA or anti-FLAG agarose as described in^46^. Imaging of isolated proteins was performed using ‘tapping’ mode in air, as described previously (23). Nanoscope software was used to manually determine particle heights and diameters and used to calculate the molecular volume of each particle using the equation:

1 $${V}_{m}=(\pi h/6)\,(3{r}^{2}+{h}^{2})$$

where *h* is the particle height and *r* is the radius^47^. This equation assumes that the adsorbed particles adopt the form of a spherical cap. Molecular volume based on molecular mass was calculated using the equation:

2 $${V}_{c}=({M}_{0}/{N}_{0})({V}_{1}+d{V}_{2})$$

where *M*_0_ is the molecular mass, *N*_*0*_ is Avogadro’s number, *V*_1_ and *V*_2_ are the partial specific volumes of particle (0.74 cm^3^/g) and water (1 cm^3^/g), respectively, and *d* is the extent of protein hydration (taken as 0.4 g water/g protein).

### Statistical analysis

Data are presented as mean ± SEM as indicated in the figure legends. Differences between groups were analysed with ANOVA followed by a Tukey’s post-hoc test or Dunnett’s multiple comparison post-hoc test as indicated. P < 0.05 was considered statistically significant.

## Supplementary information

Supplementary Figures.

## Data Availability

The datasets generated during and/or analysed during the current study are available from the corresponding author on reasonable request.
