# Supplementary material for: Oligomers of the lipodystrophy protein seipin may co-ordinate GPAT3 and AGPAT2 enzymes to facilitate adipocyte differentiation
Source: Sci Rep. 2020 Feb 24;10:3259. doi: 10.1038/s41598-020-59982-5 (PMC7039881; doi:10.1038/s41598-020-59982-5)
Supplement: Supplementary file 1 — Supplementary Figures. [file 41598_2020_59982_MOESM1_ESM.pdf]

Supplementary Information

Oligomers of the lipodystrophy protein seipin may co-ordinate GPAT3 and AGPAT2 enzymes to facilitate adipocyte differentiation.

M.F. Michelle Sim[1,4], Elisa Persiani[2,4], Md. Mesbah Uddin Talukder[3], George D. Mcilroy[2], Ahlima Roumane[2], J. Michael Edwardson[3] and Justin J. Rochford[1,2*]

[1]University of Cambridge Metabolic Research Laboratories, Institute of Metabolic Science, Addenbrooke's Hospital, Cambridge, CB2 0QQ, UK.

[2]Rowett Institute and the Aberdeen Cardiovascular and Diabetes Centre, University of Aberdeen, Foresterhill, Aberdeen, AB25 2ZD, UK.

[3]Department of Pharmacology, University of Cambridge, Cambridge CB2 1PD, UK.

Sim et al. Supplementary Figure 1

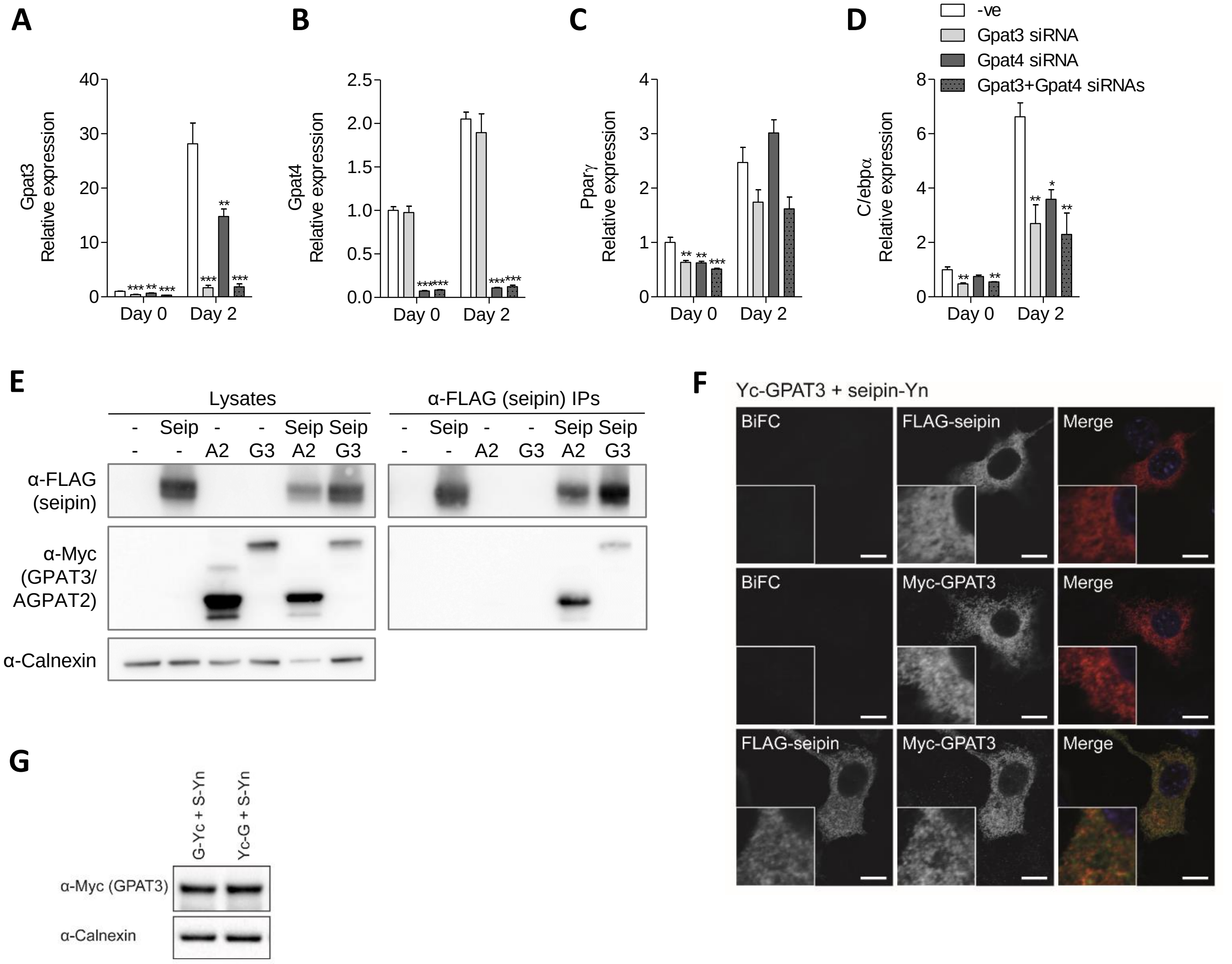

Figure S1. Supplementary Data. C3H10T1/2 cells were transfected with control siRNA (-ve) or siRNA targeting GPAT3, GPAT4 or both GPAT3 and GPAT4, as indicated, at day -2 and day 0 of differentiation. RNA was extracted at day 0 or day 2 of differentiation, and expression of Gpat3 (**A**), Gpat4 (**B**), Pparγ (**C**) and C/ebpα (**D**) was determined by qPCR. Data are presented as means ± SEM, (n=3), normalized to Ywhaz and Nono. Statistical analysis was made by one-way ANOVA followed by Tukey's multiple comparison post-hoc test. Statistically significant differences are indicated by * $p<0.05$, ** $p<0.01$, *** $p<0.001$. (**E**) HEK293 cells were transfected with Myc-AGPAT2 (A2) or Myc-GPAT3 (G3) in the absence or presence of FLAG-seipin (Seip) as indicated. Lysates and anti-FLAG immunoprecipitates were separated by SDS-PAGE and immunoblotted with antibodies to FLAG or Myc to detect tagged proteins or calnexin as a loading control. (**F**) 3T3-L1 cells were induced to differentiate, and immediately co-transfected with Yc-Myc-GPAT3 and FLAG-seipin-Yn. Following a temperature shift to induce the formation of any reconstituted YFP, cells were fixed on day 3 of differentiation and stained for FLAG-seipin (top panels) or Myc-GPAT3 (middle panels) and DAPI to label nuclei. The lack of direct interaction between seipin-Yn and Yc-GPAT3 is indicated by the absence of a YFP signal (in contrast to that seen in Fig. 1F). Identically-transfected cells were co-immunostained for FLAG-seipin-Yn and Yc-Myc-GPAT3 (bottom panels). Individual images are shown in grayscale and merged images show overlay of YFP (yellow) with FLAG-seipin (red) or Myc-GPAT3 (red). Bottom panels, individual images are shown in grayscale and merged image shows overlay FLAG-seipin (green) and Myc-GPAT3 (red). Inset boxes show zoomed images. Scale bars, 10 µm. (**G**) Representative immunoblot of cell lysates as transfected in (E) and in (Fig. 1F), using anti-Myc antibody to detect GPAT3-Yc and Yc-GPAT3 expression, and showing calnexin as a loading control.

Sim et al. Supplementary Figure 2

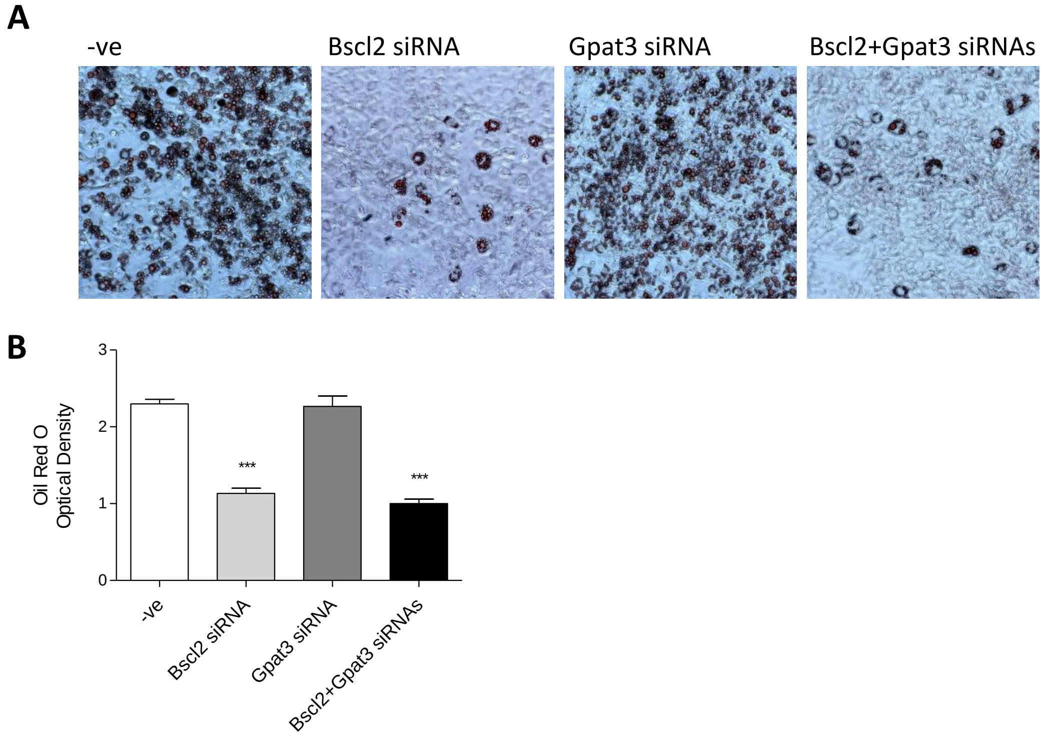

Figure S2. Supplementary Data. (**A**) C3H10T1/2 cells were transfected with control siRNA (-ve) or siRNA targeting Bscl2, Gpat3 or both Bscl2 and Gpat3, as indicated, at day -2 and day 0 of differentiation. Cells were differentiated for 10 days and oil red O staining used to visualise lipid accumulation. (**B**) Oil red O was quantified following spectrophotometric reading at 520nm. Data are presented as means ± SEM, (n=3). Statistical analysis was made with one-way ANOVA, followed by Dunnett's multiple comparison post hoc test. Statistically significant differences are indicated by *** $p<0.001$.

Sim et al. Supplementary Figure 3

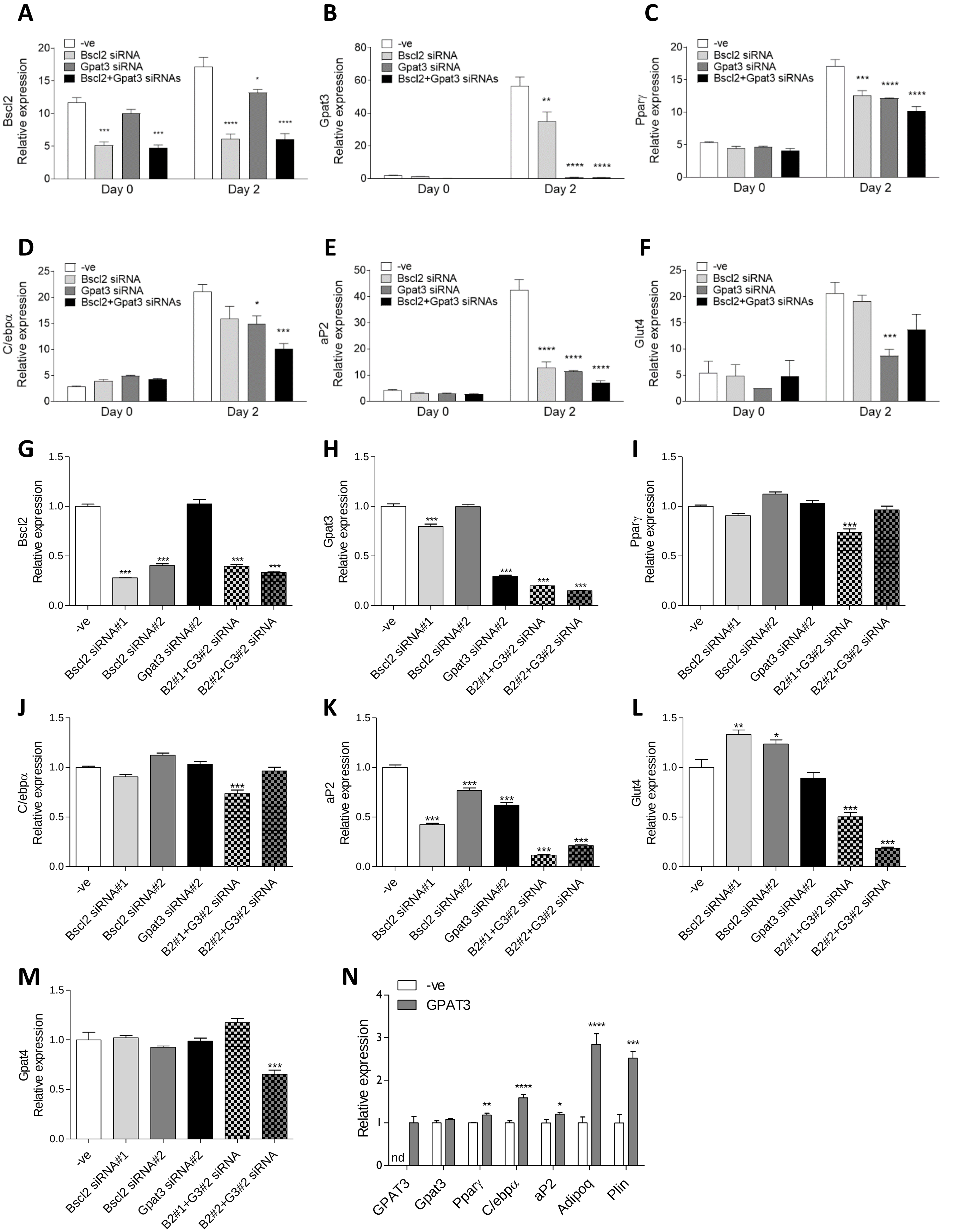

Sim et al. Supplementary Figure 3

Figure S3. Supplementary Data. 3T3-L1 preadipocytes were transfected with control siRNA (-ve) or siRNA targeting seipin (Bscl2), Gpat3, or both seipin (Bscl2) and Gpat3, as indicated, at day -2 and day 0 of differentiation. RNA was extracted at day 0 or day 2 of differentiation and expression of Bscl2/seipin (**A**), Gpat3 (**B**), Pparγ (**C**), C/ebpα (**D**), aP2 (**E**) and Glut4 (**F**) determined by qPCR. Data are presented as relative mRNA expression (means ± SEM, n=4), normalized to Ywhaz. Statistically significant differences compared to control siRNA at each time point are indicated by * $p<0.05$, ** $p<0.01$, *** $p<0.001$, **** $p<0.0001$. To examine the effect of alternative siRNA targeting Bscl2 and Gpat3, 3T3-L1 preadipocytes were transfected with control siRNA (-ve) or siRNA targeting seipin as in A-F (Bscl2siRNA#1) or an alternative sequence (Bscl2siRNA#2) alone or in combination with a second siRNA targeting Gpat3 different from that used in A-F (Gpat3siRNA#2), as indicated, at day -2 and day 0 of differentiation. RNA was extracted at day 2 of differentiation and expression of Bscl2/seipin (**G**), Gpat3 (**H**), Pparγ (**I**), C/ebpα (**J**), aP2 (**K**) and Glut4 (**L**) and Gpat4 (**M**) determined by qPCR. Data are presented as relative mRNA expression (means ± SEM, n=4), normalized to Ywhaz. Statistically significant differences compared to control siRNA at each time point are indicated by * $p<0.05$, ** $p<0.01$, *** $p<0.001$. (**N**) 3T3-L1 cells were transfected with empty vector (-ve) or human GPAT3 as indicated at day -2 and day 0 of differentiation. RNA was extracted at day 2 of differentiation and expression of human GPAT3, endogenous murine Gpat3, Pparγ, C/ebpα, aP2, adiponectin (Adipoq) and perilipin (Plin) determined by qPCR. Data are presented as relative mRNA expression (means ± SEM, n=6), normalized to the geometric mean of three stable reference genes (Nono, Ywhaz and Hprt). Statistically significant differences compared to empty vector transfected cells are indicated by * $p<0.05$, ** $p<0.01$, *** $p<0.001$, **** $p<0.0001$.

Sim et al. Supplementary Figure 4

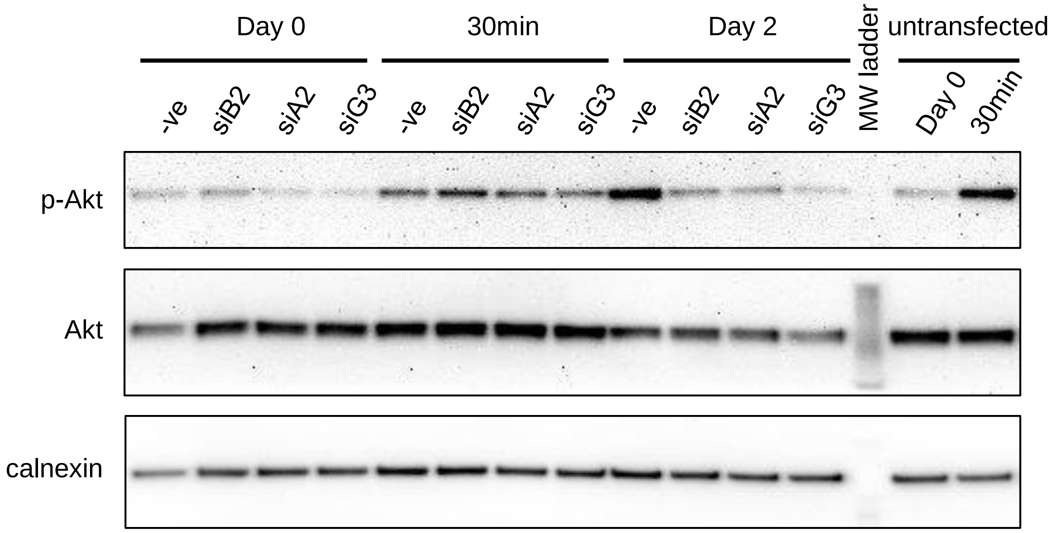

Figure S4. Supplementary Data Related to Fig. 6G. Full blots from which images in Fig. 6G were taken. C3H10T1/2 cells were transfected with control siRNA (-ve) or siRNA targeting seipin (siB2), Agpat2 (siA2) or Gpat3 (siG3) as indicated at day -2 and day 0 of differentiation. Cells were either lysed at Day 0 (prior to transfection at this time point) or transfected and induced to differentiate for 30 minutes or 2 days prior to lysis. Lysates were then analysed for total (Akt) and phospho-Akt (p-Akt) levels by immunoblotting as indicated. Untransfected cells were also lysed at day 0 and following 30 minutes of differentiation. We found that the transfection at day 0 significantly impaired the acute induction of Akt phosphorylation and so used only the Day 0 and Day 2 data in the analyses shown in Fig. 6G and H. Blots are representative of 3 independent replicate experiments. Calnexin was used as a loading control.
